# Supplementary material for: Deep-sea hiatus record reveals orbital pacing by 2.4 Myr eccentricity grand cycles
Source: Nat Commun. 2024 Mar 12;15:1998. doi: 10.1038/s41467-024-46171-5 (PMC10933315; doi:10.1038/s41467-024-46171-5)
Supplement: Supplementary file 1 — Supplementary Information [file 41467_2024_46171_MOESM1_ESM.pdf]

Deep-sea hiatus record reveals orbital pacing by 2.4 Myr eccentricity grand cycles

Adriana Dutkiewicz[1*], Slah Boulila[2,3], and R. Dietmar Müller[1]

[1]EarthByte Group, School of Geosciences, The University of Sydney, Sydney NSW 2006, Australia

[2]Sorbonne Université, CNRS, Institut des Sciences de la Terre Paris, ISTeP, 75005 Paris, France

[3]ASD/IMCCE, CNRS-UMR8028, Observatoire de Paris, PSL University, Sorbonne Université, 75014 Paris, France.

*Corresponding author : adriana.dutkiewicz@sydney.edu.au

Supplementary information

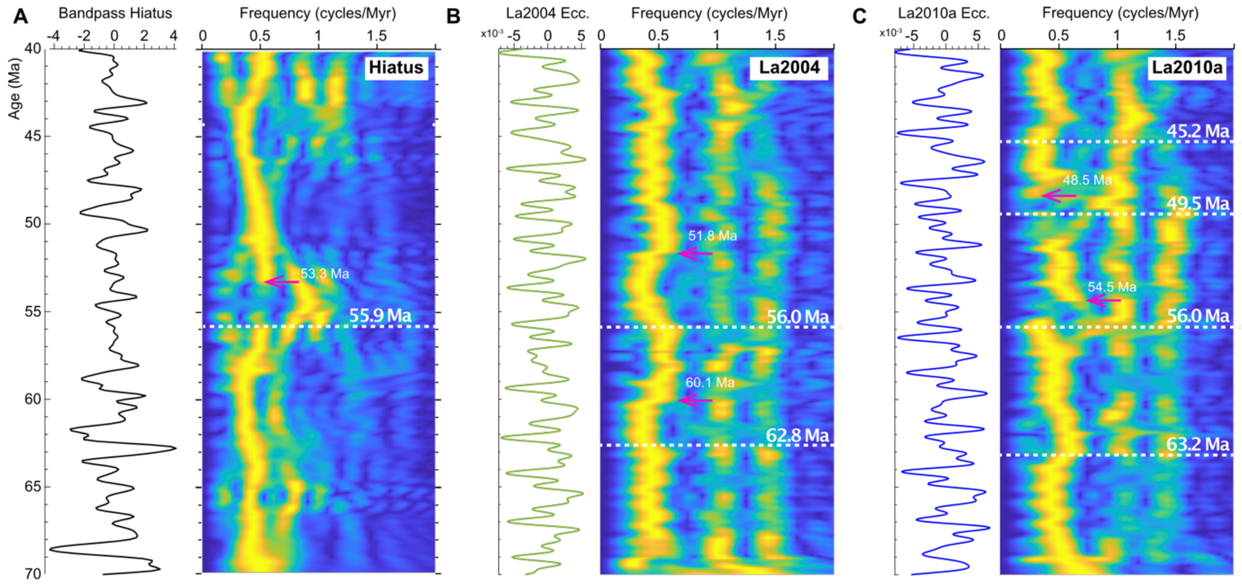

Fig. S1. Analysis of low-frequency variations in hiatus-frequency and Earth's orbital eccentricity data. (A) Taner bandpass of hiatus-frequency data (cutoff frequencies: 0.28 and 1.8 cycles/Myr, Taner filter roll-off = 10^{20}), along with evolutive FFT spectrogram (window = 6 Myr, step = 0.1 Myr). (B, C) Taner lowpass La2004 and La2010a eccentricity data (cutoff frequencies: 0 and 1.8 cycles/Myr, Taner filter roll-off = 10^{20}), along with evolutive FFT spectrogram (window = 6 Myr, step = 0.1 Myr).

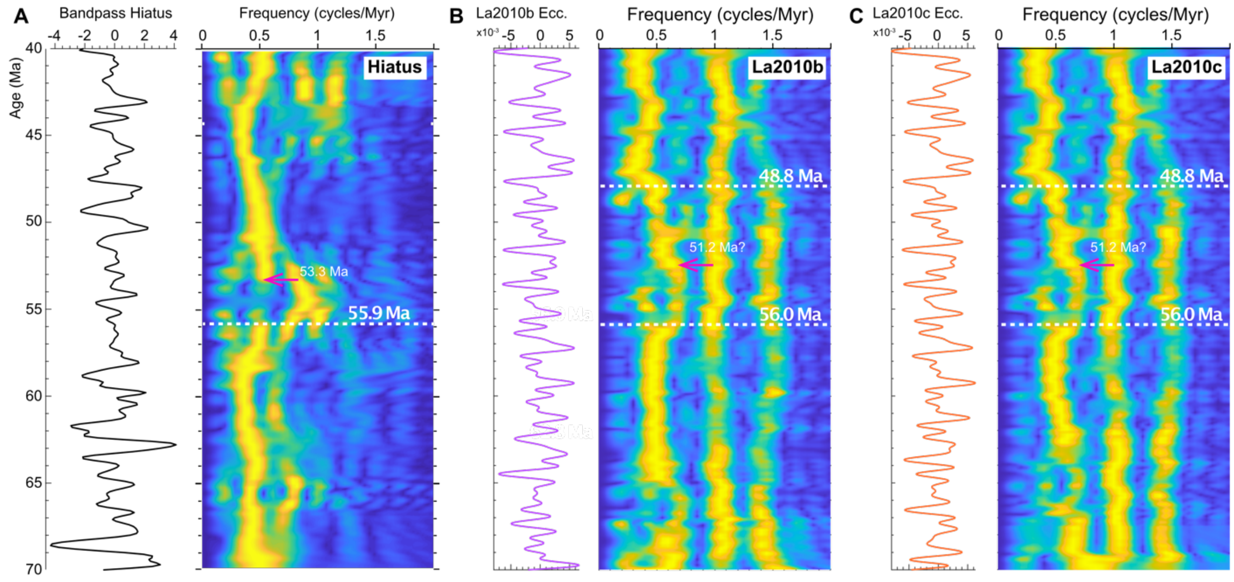

Fig. S2. Analysis of low-frequency variations in hiatus-frequency and Earth's orbital eccentricity data. (A) Taner bandpass of hiatus-frequency data (cutoff frequencies: 0.28 and 1.8 cycles/Myr, Taner filter roll-off = 10^{20}), along with evolutive FFT spectrogram (window = 6 Myr, step = 0.1 Myr). (B, C) Taner lowpass La2010b and La2010c eccentricity data (cutoff frequencies: 0 and 1.8 cycles/Myr, Taner filter roll-off = 10^{20}), along with evolutive FFT spectrogram (window = 6 Myr, step = 0.1 Myr).

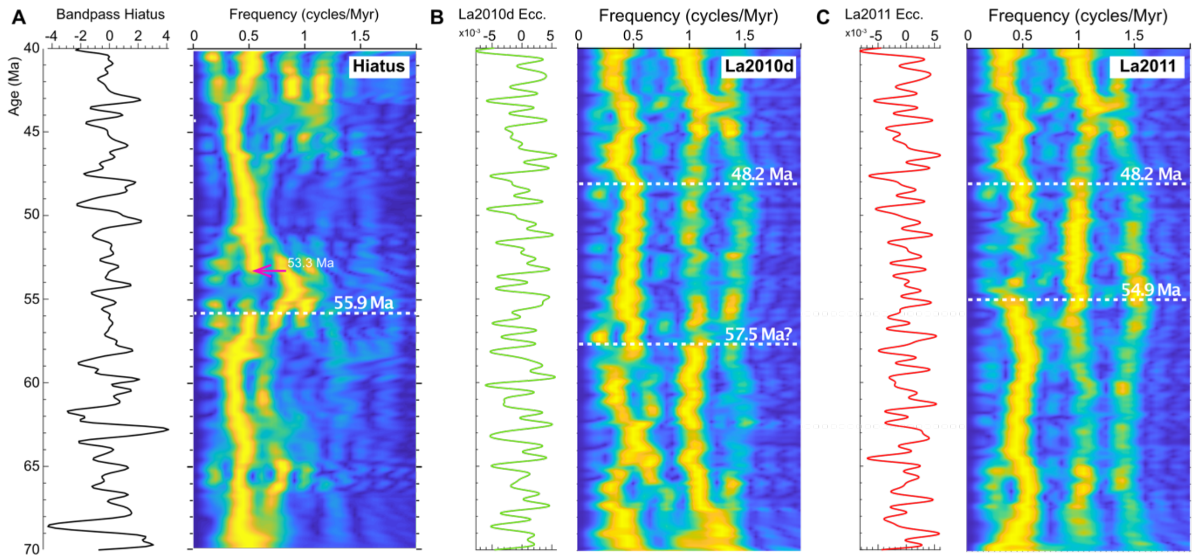

Fig. S3. Analysis of low-frequency variations in hiatus-frequency and Earth's orbital eccentricity data. (A) Taner bandpass of hiatus-frequency data (cutoff frequencies: 0.28 and 1.8 cycles/Myr, Taner filter roll-off = 10^{20}), along with evolutive FFT spectrogram (window = 6 Myr, step = 0.1 Myr). (B, C) Taner lowpass.

Table S1. Main parameters of the astronomical solutions used in this study.

Astronomical solution	Initial conditions	Time spans of initial conditions (kyr)	Step size of numerical integration (yr)	Effect of five major asteroids
La2004 (ref. 5)	DE406 (ref. 66)	-5 to +1	5×10^{-3}	Not included
La2010a (ref. 6)	INPOP08a (ref. 68)	0 to 580	10^{-3}	Included
La2010b (ref. 6)	INPOP08a (ref. 68)	0 to 580	5×10^{-3}	Included
La2010c (ref. 6)	INPOP08a (ref. 68)	0 to 1000	5×10^{-3}	Not included
La2010d (ref. 6)	INPOP06 (ref. 69)	0 to 1000	5×10^{-3}	Included
La2011 (ref. 20)	INPOP10a (ref. 70)	0 to 1000	5×10^{-3}	Included
